# Phytosterols and the Digestive System: A Review Study from Insights into Their Potential Health Benefits and Safety

**DOI:** 10.3390/ph17050557

**Published:** 2024-04-26

**Authors:** Edyta Miszczuk, Andrzej Bajguz, Łukasz Kiraga, Kijan Crowley, Magdalena Chłopecka

**Affiliations:** 1 Division of Pharmacology and Toxicology, Department of Preclinical Sciences, Institute of Veterinary Medicine, Warsaw University of Life Sciences, Ciszewskiego 8, 02-786 Warsaw, Poland; edyta_miszczuk@sggw.edu.pl (E.M.); kijan_crowley@sggw.edu.pl (K.C.); 2 Department of Biology and Plant Ecology, Faculty of Biology, University of Bialystok, Ciołkowskiego 1J, 15-245 Bialystok, Poland; abajguz@uwb.edu.pl

**Keywords:** phytosterols, β-sitosterol, stigmasterol, digestive system, anti-inflammatory properties, hepatoprotective properties, anti-oxidative properties, anticancer properties, adverse effects

## Abstract

Phytosterols are a large group of substances belonging to sterols—compounds naturally occurring in the tissues of plants, animals, and humans. The most well-known animal sterol is cholesterol. Among phytosterols, the most significant compounds are β-sitosterol, stigmasterol, and campesterol. At present, they are mainly employed in functional food products designed to counteract cardiovascular disorders by lowering levels of ‘bad’ cholesterol, which stands as their most extensively studied purpose. It is currently understood that phytosterols may also alleviate conditions associated with the gastrointestinal system. Their beneficial pharmacological properties in relation to gastrointestinal tract include anti-inflammatory and hepatoprotective activity. Also, the anti-cancer properties as well as the impact on the gut microbiome could be a very interesting area of research, which might potentially lead to the discovery of their new application. This article provides consolidated knowledge on a new potential use of phytosterols, namely the treatment or prevention of gastrointestinal diseases. The cited studies indicate high therapeutic efficacy in conditions such as peptic ulcer disease, IBD or liver failure caused by hepatotoxic xenobiotics, however, these are mainly in vitro or in vivo studies. Nevertheless, studies to date indicate their therapeutic potential as adjunctive treatments to conventional therapies, which often exhibit unsatisfactory efficacy or serious side effects. Unfortunately, at this point there is a lack of significant clinical study data to use phytosterols in clinical practice in this area.

## 1. Introduction

Phytosterols are a large group of naturally occurring plant-derived steroids with chemical similarities to mammalian sterols such as cholesterol. Being elements of cell membranes and intracellular organelles, they are present in all plant tissues. They help stabilize phospholipid bilayers and function as signalling molecules acting on receptors for steroid hormones. Their role is to adapt plants to low temperatures and mitigate the negative effects of pathogens on them [1]. Phytosterols are found in foods of plant origin, especially in oils, grains and nuts [2]. The concentration of phytosterols in vegetable oils is estimated at 0.1 to 1.0% [3]. A typical Western human diet provides about 200–300 mg of phytosterols per day [4]. In turn, stanols are hydrogenated phytosteroids found in some man-made dietary products, like margarine. Such dietary products that have a documented positive effect on the human body in addition to the nutritional effect they exhibit are termed “functional foods”. The best known biological function of phytosterols is their role in the prevention of cardiovascular diseases by lowering the level of the low-density lipoprotein (LDL) and very-low-density lipoprotein (VLDL) fractions of cholesterol [5]. However, it should be emphasized that the results of many studies indicate that phytosterols can affect various organism functions, including digestive system.

Various factors, such as the Western diet, stress, autoimmunity, certain drugs or pathogens influence diseases associated with functions of digested system. These factors, particularly improper nutritional habits, have a significant impact on pathologies such as inappropriate motor activity of gastrointestinal tract (GIT), gastric ulcers, inflammatory bowel disease (IBD) [6], as well as liver and pancreatic diseases [7]. Current drug treatment for these diseases is inadequate and is becoming increasingly problematic, due to the side effects it causes, among other reasons [8]. In this case, clinicians and researchers are trying to find a solution in traditional supplements, claiming that a diet rich in phytosterols is the new nutritional postulate. Considering the known and constantly discovered biological properties of phytosterols, it seems reasonable to determine the possibility of their use as an adjunctive treatment to conventional pharmacotherapy for GIT diseases, including GI motility disorders, peptic ulcers, inflammatory and auto-immune diseases of the intestine, as well as pancreatic and liver conditions.

The article aims to review a wide range of publications examining the effects of phytosterols on the digestive system, including the GIT and accessory organs such as the liver. In addition to describing the most common uses of phytosterols, another goal of the paper was to demonstrate their potential as adjunctive treatments to conventional therapies. It should be noted that the multitude of studies in this area are preclinical, both in vitro and in vivo, although their results indicate great potential for use in clinical trials, of which very few have been conducted at this point.

This review may improve our understanding of the potential use of phytosterols in pathological conditions of the gastrointestinal tract, as well as contribute to the discussion on their safety.

## 2. Chemical Properties of Phytosterols

**Figure 1:**
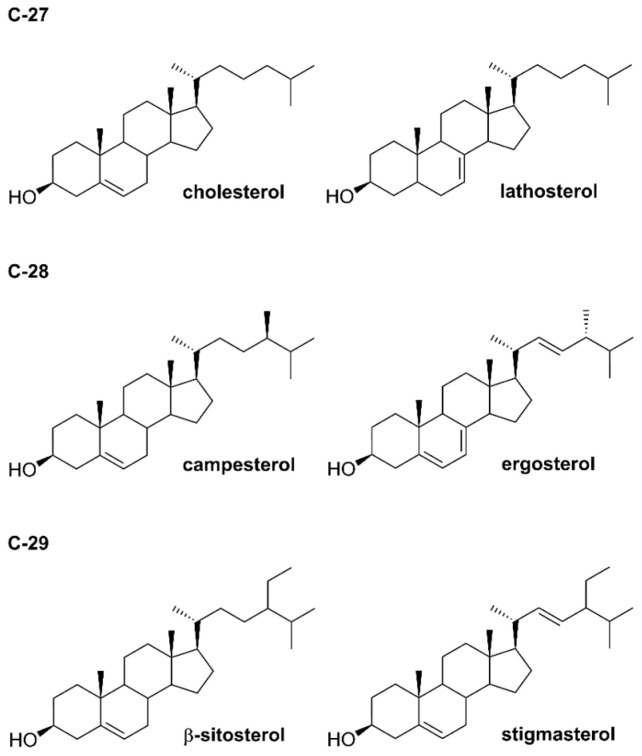
Structures of selected phytosterols.

Phytosterols were described chemically for the first time in 1922 [9] and to date, more than 250 compounds have been isolated [10]. They belong to the triterpene family with a tetracyclic cyclopenta-α-phenanthrene ring. Phytosterols are derivatives of C-28 (e.g., campesterol) and C-29 (e.g., β-sitosterol and stigmasterol) sterols in contrast to cholesterol, which belongs to compounds of C-27 [11]. Phytostanols, including β-sitostanol, and campestanol, are characterized by the lack of the Δ^5^ double bond on the B-ring and they contain an additional methyl or ethyl group linked to the cholesterol aliphatic side carbon chain (Figure 1). It seems that β-sitosterol, stigmasterol and campesterol are the main phytosterols of plant tissues [12,13].

### Absorption, Distribution and Excretion of Phytosterols

The absorption of phytosterols depends on the side chain, the applied form, and the chemical structure. Less than 5% of phytosterols are absorbed orally, in contrast to cholesterol, 15–80% of which is absorbed. Campesterol is better absorbed than β-sitosterol, while stigmasterol is the least absorbed [14]. They are structurally similar to cholesterol and compete with the absorption of this sterol in the gut, eventually decreasing endogenous production of the LDL fraction [15,16]. The phenomenon of inhibiting cholesterol’s absorption mediated by phytosterols has been studied since the 1950s [15]. Organisations such as the American Heart Association and European Dietary Guidelines recommend the use of phytosterols as primary or secondary agents in treating hypercholesterolemia [17]. There are three pathways of cholesterol elimination. First is the replacement of cholesterol in lipid micelles, which causes a higher amount of cholesterol to be excreted in the faeces [18]. The second mechanism (by which phytosterols lower circulating cholesterol level) is excretion of cholesterol into the digestive tract by enterocytes. Transport through the enteric cell membrane is affected by the Niemann-Pick C1-Like 1 protein. After that, the lipid micelle is excreted by efflux transporters from the ABC (ATP-Binding Cassette) transporter family [19]. This type of transmembrane proteins performs active transport of various substances across cellular membranes, mainly toxic metabolites. The proteins allowing efflux of phytosterols and cholesterol are ABCG5 and ABCG8 [20]. According to the third mechanism, a small amount of phytosterols are incorporated into chylomicrons, which are captured by the liver and then excreted with bile through ABCG5 and ABCG8 transporters [21].

However, in some cases phytosterols are associated with certain abnormalities, as discussed in the following section.

## 3. Phytosterol-Associated Abnormalities

### 3.1. Phytosterolemia

A significant aspect concerning cholesterol regulation is a serious, but very rarely occurring disease—sitosterolemia, also known as phytosterolemia. This is a genetic autosomal recessive disorder that increases intestinal absorption and decreases biliary excretion of dietary sterols, causing hypercholesterolemia, and premature coronary atherosclerosis that can even lead to death. Sitosterolemia was described for the first time in 1974 by two sisters of Swiss and German origin [22]. It is caused by mutations of the genes encoding the structure and function of segments of the ABC cassette—more detailly in the ABCG5 (sterolin-1) and ABCG8 (sterolin-2) which are expressed in the liver and intestine [23]. Moreover, patients with phytosterolemia have high cholesterol synthesis due to HMG-CoA reductase upregulation. People suffering from this disease absorb phytosterols, but they are not able to excrete them in the gut. Patients absorb 15% to 60% of the ingested sitosterol (contrary to healthy persons absorbing approximately 5%), leading to abnormally increased levels of phytosterols and 5α-saturated stanols in blood plasma [19], thereby blood sterol levels are estimated to be over 30 times higher [24]. Plant sterols, as well as animal sterols like cholesterol, tend to accumulate in tissues [25]. Sitosterolemia manifests with aberrations in blood tests, such as thrombocytopenia or macrothrombocytopenia. However, the main symptom of sitosterolemia is the formation of xanthomas around the knees, elbows, palms, or eyes. For sitosterolemia, the recommended treatment is a restriction in foods containing phytosterols, such as nuts, seeds, oils, and some shellfish, such as clams and oysters. In specific therapy, cholesterol absorption inhibitors such as cholestyramine are used [19]. Other authors point to ezetimibe therapy, which reduces phytosterol absorption in the gastrointestinal tract and its metabolism, what eventually causes a lower ratio of phytosterol in plasma [23,26].

### 3.2. Intestinal Failure-Associated Liver Disease (IFALD)

In the case of infants and children, parenterally administered fat emulsions may cause serious diseases such as intestinal failure-associated liver disease (IFALD). Lipid emulsions contain high amount of phytosterols, which can promote IFALD. Other components are polyunsaturated fatty acids and tocopherols. The link between IFALD and soybean fat emulsions was discovered in 1982 [27]. In the pathogenesis of IFALD, phytosterols reduce bile flow and inhibit bile secretion. In the molecular biology research on IFALD, it was discovered that phytosterols disrupt bile acid balance. One of the phytosterols, stigmasterol, inhibits the expression of the farnesoid X receptor—the key receptor involved in the regulation of bile acids synthesis, causing liver damage. Intravenous administration of soybean fat emulsions may also cause the accumulation of phytosterols in the organism, as well as dysbiosis and intestinal barrier dysfunction. The disease is manifested by an elevated bilirubin level, greater than 2 mg/dL [28]. The symptoms of IFALD are liver steatosis and cholestasis at the beginning of the disease, leading to fibrosis and cirrhosis.

### 3.3. Interactions in Absorption of Vitamins

Although phytosterols and functional foods such as margarine containing phytosterols and stanols have many positive aspects, some experimental data suggests that they can disrupt the absorption of fat-soluble vitamins from the lumen of the gastrointestinal tract. Experiments were conducted on Caco-2 cell lines demonstrating impaired absorption of β-carotene and vitamin D in the presence of phytosterols [12,21,29]. Other studies claim, that although phytosterols decrease the uptake of β-carotene and vitamin D, they do not interfere with vitamin E and K absorption [12,30]. β-carotene malabsorption can be easily improved by the increased consumption of colourful fruits and vegetables [21]. Several studies showed that increasing intakes of carotenoid-rich fruits and vegetables would prevent phytosterol-induced decreases in plasma carotenoid concentrations [31].

However, in most cases, intake of phytosterols is associated with beneficial health effects. Most significant seems to be their ability to lower cholesterol levels and contribute to the treatment of gastrointestinal diseases. Of importance are also their anti-inflammatory and hepatoprotective properties. There are also reports of their anticancer activity, but this point is debatable. In cases of liver and pancreatic insufficiency or gastrointestinal diseases, including peptic ulcers and IBD, traditional pharmacology is inadequate or causes side effects in many patients, therefore it appears that phytosterols could be an effective treatment to support conventional therapies. However, phytosterol research is still in the preclinical in vitro or in vivo phase and has typically been conducted on mouse/rat models. The aspects of the health-promoting effects of phytosterols, as well as their potential clinical application in GIT diseases treatment, are described in the following section.

## 4. Health-Promoting Effects of Phytosterols

### 4.1. Anti-Inflammatory Properties

#### 4.1.1. Anti-Ulcerogenic

In the pathogenesis of inflammation, two isoenzymes of cyclooxygenases (COX): COX-1 and COX-2 have a pivotal function. COX-1 plays an important role exhibiting cytoprotective properties mainly in the stomach and intestines, and contributes to blood clotting. In contrast, COX-2 is crucial to the inflammatory response and is found in tissues in pathological state of inflammation, mediating pro-inflammatory cytokines synthesis from arachidonic acid. A rarely mentioned property of phytosterols is their ability to selectively inhibit the activity of COX-2, the pro-inflammatory isoenzyme. This function was proven in research conducted by Akinloye, et al. [32] using phytosterol extract from tobacco plant (*Nicotiana tabacum*). They administered isolated compounds from *N. tabacum* to Wistar rats and performed a histological analysis and reverse transcription-polymerase chain reaction of the samples collected from organs subjected to HCl and ethanol, which induced their inflammation. It was observed that phytosterols down-regulated COX-2 mRNA expression and created hydrogen bonds that selectively supressed COX-2 activity, as well as regenerated parietal cells and the gastrointestinal architecture. Moreover, it was observed that phytosterols also support the liver’s antioxidant defence. Importantly, the study concluded that phytosterols are efficient in decreasing inflammation, they are safe and have good oral bioavailability [32]. Some papers on phytosterols, such as β-sitosterol, and its glycosides and esters demonstrated their role in preventing and healing stomach and duodenal ulcer disease [33]. In contrast to non-steroidal anti-inflammatory drugs (NSAIDs), phytosterols inhibit only one of the COX family isoenzyme, namely COX-2. Consumption of foods with a large amount of phytosterols in developing countries, such as those from Southeast Asia, helps prevent the development of gastric and duodenal ulceration [34].

**Figure 2:**
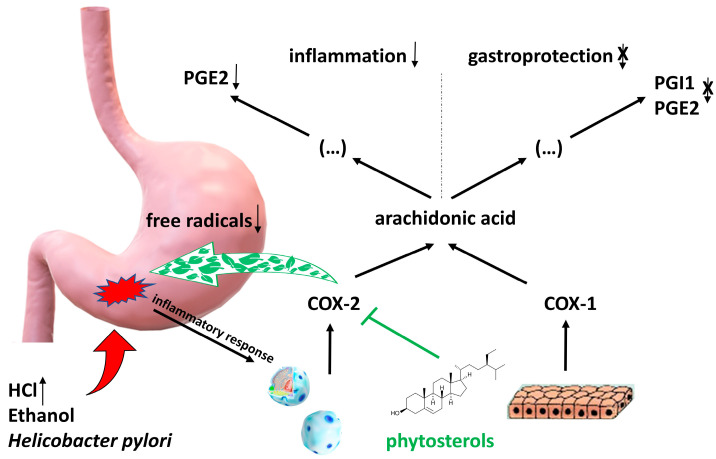
**Mechanism of anti-ulcer action of phytosterols.** As a result of damage to the gastric mucosa by factors such as ethanol, *Helicobacter pylori* or under the influence of reduced pH due to excessive activity of the parasympathetic system, there is a release of pro-inflammatory factors that activate leukocytes for increased synthesis of cyclooxygenase 2 (COX-2). Phytosterols selectively block cyclooxygenase 2 (COX-2), which leads to inhibition of the synthesis of prostaglandin PGE2 from arachidonic acid, known to be a potent mediator of inflammation within damaged tissues. Constitutively synthesized by epithelial cells cyclooxygenase 1 (COX-1) is not inhibited by phytosterols, so the synthesis of PGE2 and PGI1, which act within the mucosa as gastroprotective (cytoprotective) factors, is not reduced. Another mechanism is the decrease in oxidative stress due to the lowering of free radical production.

Rats exposed to aspirin (acetylsalicylic acid), ethanol, and fed a diet rich in phytosterols for 2 weeks showed the supportive and protective role of phytosterols in inhibiting the development of inflammation and further development of ulcers [35]. *Cissus quadrangularis* (Vitaceae) extract containing β-sitosterol and ketosterol showed gastric mucosa protection properties by lowering free radical production when taking aspirin and indomethacin—an NSAIDs [36]. Phytosterols have a soothing effect on ulcers caused by *Helicobacter pylori* or NSAIDs, such as aspirin [31,35]. In a study conducted by Tovey [31], phytosterols isolated from *Dolichos biflorus* were tested on a rat model. The fraction of isolated phytosterols consisted of stigmasterol and β-sitosterol, among others. They demonstrated significant effects in protecting the lower duodenal tract in different models of gastric and duodenal ulcer disease in rats. The author claims that phytosterols are very valuable in the healing of not only aspirin, ethanol, and *H. pylori*-induced ulcers but also these caused by cysteamine. This anti-inflammatory action may be also associated with phytosterols’ ability to regulate membrane fluidity and permeability by reducing proton and sodium ion leakage from cell membranes. Although there are not many papers on the role of phytosterols in ulcer treatment, this may be a rich field to explore their mechanisms and application in medicine. The mechanism of anti-ulcer action of phytosterols is presented in Figure 2.

#### 4.1.2. Inflammatory Bowel Disease Benefits

Due to the anti-inflammatory properties of phytosterols, they might play a crucial role in IBD treatment. IBD is an idiopathic chronic autoimmune disease. The inflammation occurring during this illness affects the mucosa of the digestive tract. Symptoms of IBD are weight loss, gastric disorders, diarrhea, GI bleeding and abdominal pain [37,38]. IBD generally manifests in two forms: Crohn’s disease and ulcerative colitis. In Crohn’s disease, inflammation predominantly occurs in the terminal ileum and colon, with potential involvement of any region of the GIT and may produce transmural inflammation. Conversely, ulcerative colitis primarily impacts the mucosa and submucosa of the colon, often targeting the rectum [38]. This disease is difficult to treat and even to diagnose. The main factors that contribute to IBD are an inappropriate diet full of saturated fats, and chronic stress. In recent years, more and more studies are emerging on the effects of phytotherapy on IBD and gastrointestinal motility disorders. The results indicate that phytosterols can improve gastrointestinal motility and help in inflammation remission. According to the research, colitis induced in mice by dextran sulfate sodium (DSS) administration, was responsive to phytosterol treatment, which inhibited the inflammatory process and improved mucosal healing [39]. In this case, phytosterols play a nutraceutical role with antioxidant properties facilitating prevention and remission of IBD [39,40,41,42]. Phytosterol treatment helps to reduce the severity of IBD but does not delay the onset of colitis [40,42]. Other research using herbal extracts of the genus *Canna* and species *Schisandra chinensis*, and *Glycyrrhiza glabra* containing phytosterols, improved the inflammatory and oxidative stress process in ulcerative colitis in mice [41,42,43]. Another study compared a high-fat diet to a diet containing low levels of fats [44]. Research was carried out on acute and chronic models of experimental colitis. In a high-fat diet, any beneficial effects of phytosterols on DSS-induced colitis were not reported. On the other hand, scientists observed beneficial effects of phytosterol treatment in a low-fat diet but not in a DSS-induced colitis mouse model. In summary, phytosterols improve histology, preserve the intestinal barrier, improve gastrointestinal motility, and relieve symptoms of IBD. However, the question arises, whether these results on mice models can be extrapolated to human and introduced to clinical treatment [44].

### 4.2. Hepatoprotective Properties

The third protective aspect of phytosterols is the support of liver during biotransformation, mainly of xenobiotics. Phytosterols can enhance therapy, for example, in the case of non-alcoholic fatty liver disease (NAFLD) and other liver conditions. The liver is a centre for various metabolic reactions related to oxidative stress caused by reactive oxygen species (ROS) and reactive nitrogen species (RNS). Many xenobiotics are hepatotoxic. This includes paracetamol (*N*-acetyl-*p*-acetaminophenol), carbon tetrachloride, antibiotics, pesticides, mycotoxins, and heavy metals, among others. Studies have shown that phytosterols and fatty acids such as omega-3 reduce the synthesis of inflammatory agents by lowering the level of pro-inflammatory enzymes (cyclooxygenase-2 and lipoxygenase) and, moreover, directly inhibit oxidative stress. Below are some cases of liver injury caused by different agents and the therapeutic role of phytosterols in these regard [45,46].

#### 4.2.1. Non-Alcoholic Fatty Liver Disease

Non-alcoholic fatty liver disease (NAFLD) is the most common chronic liver disease, which affects around 25% of adults. In the course of this dysfunction, the excessive accumulation of triglycerides in hepatocytes occurs without other causes of fat accumulation, e.g., chronic excessive alcohol consumption. Consequently, it even may lead to fibrosis and liver cancer. This illness has two types: NAFLD and non-alcoholic steatohepatitis. The risk factors for NAFLD are overweight, obesity, diabetes mellitus, hypertension, hypercholesterolaemia, insulin resistance, a diet rich in fructose, and older age [47]. NAFLD sometimes occurs without significant symptoms and is detected during liver biopsy or routine blood tests. The most common symptoms of NAFLD are fatigue, general malaise, and abdominal pain [48]. The best prevention for NAFLD is a healthy lifestyle and a balanced diet. Phytosterols have been tested as a treatment for NAFLD, especially β-sitosterol and stigmasterol. They seem to be beneficial in the treatment of NAFLD due to their ability to reduction of inflammation and steatosis. They inhibit NAFLD progression by modulating oxidative pathways. Stigmasterol has a beneficial effect on the liver’s triglyceride profile. In a study on mice fed a high-fat diet that developed NAFLD, β-sitosterol reduced liver cholesterol and increased polyunsaturated fatty acids level; β-sitosterol also reduces pro-inflammatory cytokines and, in some cases, can improve the histological profile of the liver [49].

#### 4.2.2. Xenobiotics Causing Liver Damage

##### Carbon Tetrachloride

Carbon tetrachloride (CCl_4_) is a liver damage-inducing xenobiotic commonly used in in vitro and in vivo studies [45]. Research results have revealed the hepatoprotective potential of β-sitosterol, although reports of its protective activity are still limited. In vivo studies in rats showed a protective effect of β-sitosterol in chronic liver disease caused by CCl_4_. The indicators of liver damage (intracellular enzyme activity in serum, markers of oxidative stress, stage of fibrosis) were significantly lower in comparison to rats exposed to CCl_4_ alone [50]. The research conducted by Abdou, et al. [51] concerning application of free β-sitosterol in suspension and in the form of hybrid lipid-polymer nanoparticles partially confirmed the hepatoprotective effect of the phytosterol, even after exposure to CCl_4_. However, in the cited study, the administration of β-sitosterol did not protect hepatocytes from damage, as evidenced by the lack of significant differences in the level of aspartate aminotransferase (AST) and alanine aminotransferase (ALT) in serum compared to the control group (receiving only CCl_4_). Other results obtained in rat model concern the ability of β-sitosterol to inhibit CYP2E1 enzymes, which is engaged in detoxification of many xenobiotics. In studies using CCl_4_, no significant change in CYP2E1 activity was found after administration of a preparation containing β-sitosterol, contrary to control group [51].

##### Paracetamol

Paracetamol is one of the most commonly used antipyretic, analgesic, and weakly anti-inflammatory drug from the non-opioid drug group. It is used to treat fever and mild to moderate pain. Paracetamol is a safe drug for short-term use in doses of up to 4 g per day/human [52]. Higher doses of paracetamol can be toxic, in particular to liver function due to the accumulation of hepatotoxic *N*-acetyl-4-benzoquinoneimine, a strong oxidant that increases the level of free radicals in the liver, causing irreversible damage to hepatocytes [53]. It is the most commonly overdosed drug in many countries [54]. Long-term use of paracetamol causes side effects manifested by a decrease in the blood level of haemoglobin, accompanied by gastrointestinal bleeding, nausea, abdominal pain, and abnormal liver function tests results [53]. Preclinical in vivo studies on a rat model showed accelerated antioxidant cellular defence and antioxidant properties of functional foods enriched with phytosterols, such as soybeans or sesame [45]. The results revealed that the studied phytochemicals can reduce serum activity of intracellular enzymes such as AST, ALT and the level of blood urea, and bilirubin. A beneficial effect has also been demonstrated by preventing oxidative stress in the liver. The results indicate that the level of glutathione in the liver of rats fed yam or carrot and treated with paracetamol at the same time is higher in comparison to the animals treated only with paracetamol. *Commiphora kua* (Bursaceae) extract, rich in phytosterols (e.g., lophenol and lathosterol), was used in an in vivo mouse model with paracetamol-induced liver damage and showed that phytosterols can mitigate the effects of hepatotoxic substances. Histological images revealed tissue regeneration in response to the injury caused by acute inflammation. In addition, increased levels of antioxidant agents like glutathione or activity of superoxide dismutase compared to mice treated only with paracetamol has been found [55]. The methanolic extract of *Ficus religiosa* was examined in a rat model with induced liver inflammation caused by paracetamol, rifampicin and isoniazid. The obtained results indicate the beneficial effect of *F. religiosa* phytochemicals in the prevention of liver damage as indicated by the lower increase of transaminase activity and higher glutathione level in the treated group compared with control animals [56].

##### Alcohol-Induced Hepatotoxicity

Excess alcohol consumption can lead to liver, brain, pancreatic, and heart disorders [49,50,57,58] and constitutes one of the most serious health problems in the world [45]. Alcohol is metabolized in the liver by alcohol dehydrogenase and the cytochrome P450 family (CYP2E1). During this process the toxic metabolite—acetaldehyde and, especially in the case of alcoholism, reactive oxygen species (ROS) are produced, causing lipid peroxidation and chronic liver inflammation. High levels of ROS activate the NFκB pathway and mitogen-activated protein kinases (MAPK). Moreover, proinflammatory interleukins like IL-6 and TNFα are secreted, and the serum activity of the aminotransferases is elevated. Long-term excessive alcohol consumption can cause liver steatosis, hepatitis, cirrhosis, and even cancer. Since no effective treatment of hepatic injury caused by alcohol has been developed so far, new agents are constantly being sought, including phytocompounds. There are some experimental data indicating the hepatoprotective properties of phytosterols in alcohol-induced liver injury. Research on a rat model showed plants and grains such as black rice, milk thistle, *Ginkgo biloba*, or turmeric can relieve the symptoms of alcohol-induced hepatotoxicity and decrease the level of toxic acetaldehyde [48]. Many natural products and herbs have been tested in the treatment and prevention of alcoholic liver disease. Research on a male mouse model conducted by Zhang, et al. [58] showed that the extract of *Sanchezia speciosa*, rich in daucosterol (sitosterol attached to a β-D-glucopyranosyl residue), inhibits MAPK pathway and upregulates the synthesis of antioxidant agents like catalase and superoxidase dismutase. This observation is important due to the fact that the levels of these protective enzymes were reduced during exposure to alcohol. The hepatoprotective effect might arise from the protection by chaperones like the p38 protein, inhibition of phosphorylation of NFκB and upregulation of the superoxide dismutase genes, i.e., SOD1 and SOD2 [58]. Daucosterol reduced level of ROS and inhibited the inflammation process. The aforementioned phytosterol also decreased expression of CYP2E1 (the level of CYP2E1 is higher in excessive alcohol consumption) as well as aldehyde dehydrogenase 2 (ALDH2) and counteracted the accumulation of high amount of ROS. It also inhibited lipid peroxidation and lipid accumulation in the liver. Another study on a rat model, led by Chen, et al. [59] showed that β-sitosterol isolated from *Artemisia* sp., a plant used in traditional Chinese medicine, also has hepatoprotective properties. The obtained results indicated that β-sitosterol reduced the level of ROS and the inflammation process, as well as fat accumulation in the liver compared with the only-alcohol treatment group [59]. β-sitosterol reduced glutathione depletion, restored activity of antioxidant enzymes and decreased production of malondialdehyde. It rebalanced proapoptotic pathways by downregulating the expression of apoptosis genes. In research conducted by Hamada, et al. [60] mice were administered ethanol for 10 days and then treated with withafterin A (one of phytosterols found in *Withania somnifera*). The tested phytosterol lowered expression of the lipogenesis genes, reducing liver lipid accumulation. To sum up, the presented data indicate that phytosterols can counteract the consequences of alcoholic liver disease, demonstrating hepatoprotective effects by reducing levels of ROS, inhibiting lipid accumulation and peroxidation in the liver, and also restoring balance between alcohol metabolites and hepatoprotective enzymes like glutathione-related enzymes or catalase.

**Figure 3:**
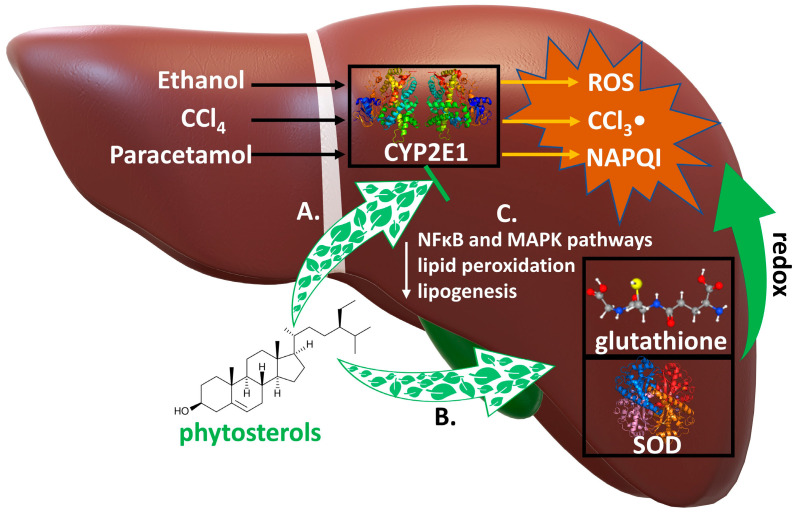
**Mechanisms of hepatoprotective action of phytosterols.** Numerous xenobiotics are metabilzed by cytochrome P450 2E1 (CYP2E1) to highly hepatotoxic compounds. Biotransformation of ethanol, CCl4, paracetamol leads to formation of very toxic metabolites: reactive oxygen species (ROS), trichloromethyl radical (CCl3-) and N-acetyl-p-benzoquinone imine (NAPQl), respectively. They are inactivated by the antioxidant compounds: glutathione and superoxide dismutase (SOD). Phytosterols: A. inhibit the expression of CYP2E1, reducing the conversion of these xenobiotics to hepatotoxic metabolites; B. increase the levels of the antioxidants glutathione and SOD, which inactivate their metabolites in redox reactions; C. inhibit NFκB and MAPK pathways, reduce the expression of the lipogenesis and lipid peroxidation genes.

Possible mechanisms of hepatotoxic action of phytosterols are indicated in Figure 3.

### 4.3. Anti-Cancer Properties

Another crucial role for phytosterols is their anti-cancer activity. Phytosterols can induce apoptosis and decrease promotion, metastasis, proliferation, and invasion of cancer cells and tissues. The anti-cancer potential of a diet rich in phytosterols can be the result of oxidative stress prevention by lowering the levels of ROS and improving immunity [61,62,63]. Results of the studies conducted on 1,2-dimethylhydrazine-initiated Wistar rats fed β-sitosterol enriched diet (isolated from *Asclepias curassavica)* showed inhibition of further cancer development without a toxic effect on healthy tissues [10]. Another study using an in vivo model conducted on Wistar rats fed with β-sitosterol showed a dose dependent reduction in crypt multiplicity with no toxic effects. The same study, among others, was conducted on an in vitro model using human adenocarcinoma cells and measuring proliferating cell nuclear antigen (PCNA) in human colon cancer cell lines (COLO 320 DM). The results showed inhibition of PCNA antigens and dose-dependent growth inhibition of COLO 320 cell lines, where the IC50 was 266.2 µM [64]. Moreover, β-sitosterol attenuated β-catenin and proliferating cell nuclear antigen expression. It also caused the cells to switch to the mitochondrial apoptosis pathway, causing a rise in the levels of ROS and calcium ions. Another study carried out on Caco-2 cells (cell lines derived from human colorectal adenocarcinoma) showed cell necrosis and inhibition of the cell cycle in the G_0_/G_1_ stage and more cells directed to the apoptosis pathway by increasing caspase levels [10,65]. Studies conducted by Álvarez-Sala, et al. [66] showed the antiproliferative role of phytosterols, which sensitized the human colon cancer cells Caco-2 and HT-29 to 5-fluorouracil treatment. They significantly raised ROS levels, activated caspase pathways, arrested cell cycles in the S stage and eventually caused apoptosis. Research conducted by Vats [67] mentions the positive role of phytosterols in colorectal cancer. Another modes of anti-tumour action shown by phytosterols is the activation of Natural Killer cells, producing higher levels of IL-2, interferon-γ and increased response from Th lymphocytes. Studies have shown that high cholesterol intake may be a cause of colorectal cancer [68]. On the other hand, intake of phytosterols with diet can prevent colon cancer by regulating intestinal microbiota, lowering the level of cholesterol metabolites [69]. The role of lowering cholesterol levels in plasma can also be supported in preventing cancer development. Scientists estimate that cancer prevention by phytosterol consumption can reach 20% [70]. Importantly, phytosterols induce the recognition of cancer cells by the immune system [70,71]. These facts show that phytosterols and a diet enriched with phytosterols can be helpful in the treatment of cancer. An important experiment was conducted in the Netherlands in the Cohort Study on Diet and Cancer. Around 120 thousand people participated in this study, where the main sources of phytosterols were bread, plant oils, vegetables, and fruits. The mean intake of all phytosterols was 285 ± 97 mg/day—this approach was based on the hypothesis that phytosterols contribute to a significant reduction in cancer incidence. However, the study found no significant association of a phytosterol-enriched diet with a lower risk of colorectal cancer [68,72]. Many studies found no significant differences in cancer prevention between the phytosterol-treated group and the placebo group, i.e., prostate, lung, colorectal, and stomach cancer [3,10]. It is worth emphasizing, that some authors claim that a high intake of stigmasterol and campesterol can lead to colorectal cancer [68] in contrast to numerous studies where phytosterols are mentioned as protective substances in the development of this type of cancer [62]. In summary, the results of studies on the effects of phytosterols on GI cancer are contradictory and require further research.

### 4.4. Impact of Gut Microbiota

The impact of gut microbiota is most noticeable in research on obese populations, with a particular focus on the analysis of cholesterol metabolites, such as coprostanol. Adjusting the balance of gut microbiota can play a crucial role in preventing a wide range of diseases, osteoporosis included [73]. Studies have demonstrated that in mice, a high-fat diet specifically influences the regulation of gut microbiota, notably increasing the presence of *Lactobacillus* and other bile salt hydrolase (BSH)-producing bacteria [72]. This observation offers insights into the mechanisms of cholesterol metabolism in mammals, showing promise for treating obesity through dietary modifications that include reducing high-fat and highly processed foods. Unabsorbed cholesterol can be biotransformed by different genera of the microorganism such as *Eubacterium* sp., *Bacteroides* sp., *Bifidobacterium* sp., to coprostanol and coprostanone, which are considered colon carcinogens. Studies utilizing in vitro gut fermentation models have shown that plant sterols, in a microbiota derived from a lean population, lead to modification of the cholesterol biotransformation pathway, resulting in decreased production of cholesterol metabolites while increasing plant sterol metabolism [69,74]. Furthermore, diets enriched with phytosterols increased the abundance of beneficial species of microbiota such as *Eubacterium halii* and reducing, at the same time, abundance of family *Erysipelotrichaceae* (Firmicutes phylum). This is most likely due to a lowering of the pH of the colon contents due to increased production of short chain fatty acids (SCFA) and the breakdown of bile acid salts into aminoacids and bile acids by BSH-producing bacteria. It should be emphasized that the presence of *Erysipelotrichaceae* species has been found in the obese human individuals, which suggests the relation of this bacterial family with metabolic disorders [69]. Additionally, in the presence of phytosterol, due to increased fermentation of dietary fiber, the production of SCFA such as acetate and butyrate significantly increased, which may be important in maintaining reduced appetite and food intake, providing energy for colonic epithelial cells and prevention of colon cancer [74,75]. Moreover phytosterols increase the diversity of bacterial genera such as *Bacteroidetes*, *Anaerostipes*, *Staphylococcus*, *Streptococcus*, and *Firmicutesin* in the breast milk, which improves its quality. It has a beneficial effect on the immune response in infants by modulating the intestinal microbiota [75].

**Figure 4:**
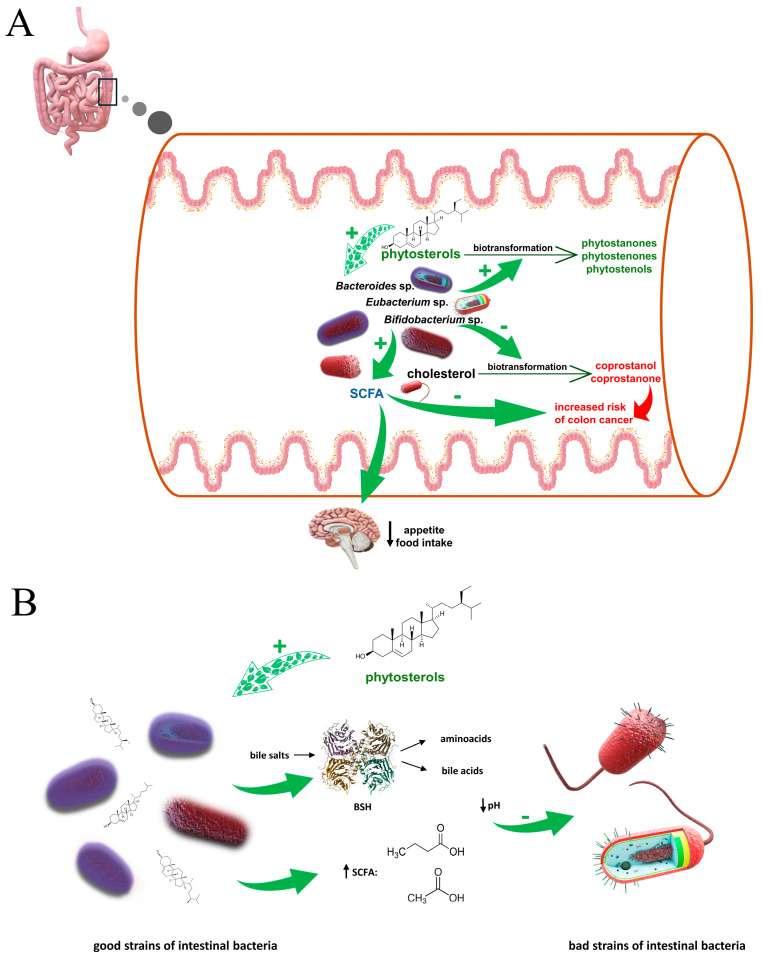
(**A**). **Influence of phytosterols on SCFA production and conversion of cholesterol, and the biological effects they produce.** Unabsorbed cholesterol is metabolised in the gut lumen by different genera of bacteria, such as *Bifidobacterium* sp., *Eubacterium* sp., *Bacteroides* sp. to metabolites which may have pro-cancerogenic effect in the colon. There are experimental data suggesting that in the presence of phytosterols the production of cholesterol metabolites is significantly reduced. Additionally, phytosterols have a positive impact on bacterial fermentation, causing an increase in SCFA production, which may be important in the treatment of patients suffering from obesity and gastrointestinal disorders. (**B**). **Effect of phytosterols on the ratio between strains of gut bacteria.** Phytosterols, in a yet unexplained way, cause upregulation of relative abundance of the good microbiota; good bacteria strains (including i.a. *Lactobacillus* and *Bifidobacterium*) produce bile salt hydrolase (BSH), which breaks down undesirable in the large intestine bile salts into amino acids and bile acids, or by fermenting dietary fiber, they increase the level of short-chain fatty acids (SCFA) (mainly butyrate and acetate); increased level of organic acids lowers pH of large intestine content which leads to decrease of bad bacteria strains abundance, including *Erysipelotrichaceae* (Firmicutes phylum). It is suggested that this bacterial family may play a role in the metabolic disorders.

The effects of phytosterols on the intestinal microbiome and associated biological effects are shown in Figure 4.

The bioactive effects of phytosterols on the digestive system in preclinical and clinical studies are summarized in Table 1.

## 5. Conclusions

The impact of phytosterols on the digestive system remains unclear and largely unexplored. Phytosterols, incorporated into functional foods like margarines, are mainly known to help prevent cardiovascular diseases by lowering cholesterol levels by reducing ‘bad’ cholesterol, which is their most documented function, with numerous articles published on this aspect. This benefit was recognized some time ago, and it is now believed that phytosterols also relieve disorders related to the digestive system. A diet enriched with phytosterols can serve as an adjunctive and supportive anti-inflammatory treatment—the studies cited in this article have shown that in in vivo models, phytosterols are effective in the treatment of gastric and duodenal ulcers and even IBD. In these pathological conditions, they contribute to reducing inflammation, accelerate regeneration of damaged tissues and normalize impaired GIT motility. Phytosterols show potential as hepatoprotective agents, acting among others as free radical scavengers. In in vivo models, they exhibit high efficacy in treating or preventing liver disease mediated by hepatotoxic xenobiotics such as ethanol, aspirin, and CCL_4_. Some studies show the effectiveness of phytosterols in the prevention of colorectal cancer by modulating the apoptosis and proliferation of cancer cells. Few studies also indicate their beneficial effect on the gut microbiome. However, the less recognized properties of phytosterols, especially concerning their anticancer or hepatoprotective roles, require more clarity. Their ability to modulate apoptosis and cell proliferation in cancer is particularly promising, though the outcomes of their impact on cancer remain inconclusive. In the context of their safe use, however, the issue of the negative effect of phytosterols on the absorption of certain vitamins and provitamins, especially vitamin D and beta-carotene is relevant. Nevertheless, the most significant problem is the almost complete lack of clinical trials, which at this point precludes their use in a clinical setting. However, a number of promising in vitro and in vivo results provide a rationale for performing such studies. Despite their known benefits, the mechanisms of action and potential applications of phytosterols also warrant further study, as they could become significant pharmacological agents in future therapies for various diseases.

## 6. Methods of Literature Search

To conduct the literature review, major scientific databases such as PubMed, Scopus and Web of Science were searched using appropriately selected keywords (including: phytosterols, campesterol, β-sitosterol, inflammation, digestive system, gastrointestinal diseases, liver peptic ulcers, inflammatory bowel disease, hepatoprotection, gastrointestinal cancer, gut microbiome, among others) and specific search criteria. The selection of articles was limited to those published within a specific time period and thematically related to the subject under discussion. In addition, citation lists of selected articles were reviewed to identify additional potential publications for inclusion in the analysis.

## Figures and Tables

**Table 1:** The bioactive impact of phytosterols on digestive system in the preclinical and clinical studies.

Model	Compound/Extract	Respective Mechanisms of Action/Effect	References
in vitro Caco-2 cell line	β-sitosterol	Directing to apoptosis pathway	[66]
in vivo Rats model	Phytosterol *Nicotiana tabacum* extract containing phytosterols	Lowering level and action of COX-2, regeneration of gastrointestinal mucosa and soothing of inflammation inhibiting mainly one isomer of COX in contrast to NSAIDs, promoting liver’s antioxidant defence	[32]
	*Hippophae rhamnoides* extract rich in β-sitosterol and its glucosides	Healing stomach and duodenal ulcers	[33]
	*Cissus quadrangularis* extract containing β-sitosterol and ketosterol	During NSAIDs therapy protecting gastric mucosa from free radicals	[36]
	*Dolichos biflorus* extract rich in stigmasterol and β-sitosterol	Preventing and treating gastroduodenal ulcers	[31]
	β-sitosterol	Hepatoprotective properties even after exposure on CCl_4_, anticancer properties without damage of healthy tissues	[10,51]
	Phytosterol enriched soybeans and sesame	Elevated antioxidant properties in liver damage caused by paracetamol	[45]
	Methanolic extract of *Ficus religiosa*	Lower increase of transaminase activity and higher glutathione level after liver damage caused by paracetamol, isoniazid and rifampicin	[56]
	β-sitosterol isolated from *Artemisia*	Increase level of glutathione, reducing level of ROS and inflammation process, decreased production of malondialdehyde	[59]
Mice model	Phytosterol extract from Canna, *Schisandra chinensis* and *Glycyrrhiza glabra*	Reduce oxidative stress, onset of colitis, relieving symptoms of IBD	[41,42,43]
	Withafterin A	Lowered expression of the lipogenesis genes, reducing liver lipid accumulation	[60]
	*Commiphora kua* extract rich in lophenol and lathosterol	Tissue regeneration paracetamol induced liver damage	[55]
	*Sanchezia speciosa* extract rich in daucosterol	Upregulates the synthesis of antioxidant agents like catalase and superoxidase dismutase, elevated level of chaperone, upregulation of synthesis of the superoxide dismutase genes, i.e., SOD1 and SOD2, soothing of inflammation process	[58]
Clinical data Colorectal cancer	Phytosterols	Prevents from developing of cancer	[62,70,71]
IFALD	Stigmasterol	Inhibits farnesoid X receptor responsible for developing IFALD	[28]
The Cohort Study on Diet and Cancer	Phytosterols	No significant data about preventing from colorectal cancer	[10,68]
NAFLD	Stigmasterol and β-sitosterol	Reduces the level of liver cholesterol and increases the levels of polyunsaturated fatty acid, reduces level of proinflammatory cytokines, improves histological picture	[49]
Colorectal cancer	Stigmasterol and campesterol	Data presenting that high intake of them can lead to colorectal cancer	[62]

## Data Availability

Data sharing is not applicable.

## Abbreviations

ABC transporter	ATP-Binding Cassette transporter
ALT	alanine aminotransferase
AST	aspartate aminotransferase
CCl_4_	carbon tetrachloride
COX	cyclooxygenases
DSS	dextran sulfate sodium
HDL	high-density lipoprotein
IBD	inflammatory bowel disease
IFALD	intestinal failure-associated liver disease
LDL	low-density lipoprotein
MAPK	mitogen-activated protein kinases
NAFLD	non-alcoholic fatty liver disease
NSAID	non-steroidal anti-inflammatory drug
ROS	reactive oxygen species
VLDL	very-low-density lipoprotein
